# Dynamic Changes in the Bacterial Community and Metabolic Profile during Fermentation of Low-Salt Shrimp Paste (Terasi)

**DOI:** 10.3390/metabo12020118

**Published:** 2022-01-26

**Authors:** Henny Helmi, Dea Indriani Astuti, Sastia Prama Putri, Arisa Sato, Walter A. Laviña, Eiichiro Fukusaki, Pingkan Aditiawati

**Affiliations:** 1 School of Life Sciences and Technology, Institut Teknologi Bandung, Jalan Ganesha No.10, Bandung 40132, Indonesia; henny-helmi@ubb.ac.id (H.H.); dea@sith.itb.ac.id (D.I.A.); sastia_putri@bio.eng.osaka-u.ac.jp (S.P.P.); 2 Department of Biology, Bangka Belitung University, Kampus Terpadu Balunijuk, Jalan Raya Balunijuk, Merawang, Bangka 33172, Indonesia; 3 Department of Biotechnology, Graduate School of Engineering, Osaka University, 2-1 Yamadaoka, Suita, Osaka 565-0871, Japan; arisa_sato@bio.eng.osaka-u.ac.jp (A.S.); fukusaki@bio.eng.osaka-u.ac.jp (E.F.); 4 Industrial Biotechnology Initiative Division, Institute for Open and Transdisciplinary Research Initiatives, Osaka University, Osaka 565-0871, Japan; 5 Microbiology Division, Institute of Biological Sciences, University of the Philippines Los Baños, Los Baños, Laguna 4031, Philippines; walavina@up.edu.ph; 6 Osaka University-Shimadzu Omics Innovation Research Laboratories, Osaka University, Osaka 565-0871, Japan

**Keywords:** low-salt shrimp paste, bacterial community, metabolite changes, high-throughput sequencing, Gas Chromatography/Mass Spectrometry

## Abstract

Low-salt shrimp paste, or terasi, is an Indonesian fermented food made from planktonic shrimp mixed with a low concentration of salt. Since high daily intake of sodium is deemed unhealthy, reduction of salt content in shrimp paste production is desired. Until now, there is no reported investigation on the bacterial population and metabolite composition of terasi during fermentation. In this study, the bacterial community of terasi was assessed using high-throughput sequencing of the 16S rRNA V3–V4 region. From this analysis, *Tetragenococcus*, *Aloicoccus*, *Alkalibacillus*, *Atopostipes*, and *Alkalibacterium* were found to be the dominant bacterial genus in low-salt shrimp paste. GC/MS-based metabolite profiling was also conducted to monitor the metabolite changes during shrimp paste fermentation. Results showed that acetylated amino acids increased, while glutamine levels decreased, during the fermentation of low-salt shrimp paste. At the start of shrimp paste fermentation, *Tetragenococcus* predominated with histamine and cadaverine accumulation. At the end of fermentation, there was an increase in 4-hydroxyphenyl acetic acid and indole-3-acetic acid levels, as well as the predominance of *Atopostipes*. Moreover, we found that aspartic acid increased during fermentation. Based on our findings, we recommend that fermentation of low-salt shrimp paste be done for 7 to 21 days, in order to produce shrimp paste that has high nutritional content and reduced health risk.

## 1. Introduction

Fermented shrimp products, such as sauces and pastes, and lacto-fermented products are widely consumed in China and Southeast Asian countries [1,2,3]. These fermented products, known by their native names in different countries, are usually used as side dishes, condiments, or main dishes [1,2,3,4]. One of the famous fermented shrimp products in China and Southeast Asia is shrimp paste. It has high nutritional value because it contains amino acids, proteins, carotenoids, chitins, unsaturated fatty acids, and minerals, such as calcium, sodium, and phosphorus [4,5,6]. In fact, shrimp paste products, such as belacan and kapi, have high glutamic acid, aspartic acid, and leucine content [5,6]. Aside from its high nutritional value, shrimp paste has also been reported to exhibit antioxidant properties [4,5,7,8].

Most studies on shrimp paste from regions in China and Southeast Asia have been conducted on high-salt shrimp paste products that are fermented for more than a month [2,5,8,9,10,11]. Terasi, a traditional fermented shrimp paste from Indonesia, is widely used as a main dish or food ingredient to enhance flavor. It is generally fermented for a short time, varying from 1–4 weeks [12], unlike other shrimp paste products, such as kapi from Thailand and bagoong or alamang from the Philippines [5,8]. Traditional shrimp pastes are fermented using 25–30% salt [11], while other shrimp pastes, such as belacan, is made with a 13–17% salt concentration [1]. Meanwhile, terasi is produced with various levels of salt contents, ranging from 0% to 30%, with 5–20% being the most commonly used; good quality terasi is made using 10% [13] or 15% salt concentration [14].

The technology used for shrimp paste processing is simple and low cost, which uses a combination of salting, drying, and natural fermentation [1,11,15]. Addition of salt is widely used in the food industry, including shrimp paste or sauce production, due to its preservative properties [16,17] However, high salt content in shrimp paste, shrimp sauce, or other fish products has limited nutritional value [11,18] and high health risk, if consumed in large quantities [16,17]. Thus, the use of low-salt (NaCl) in the food product industry is becoming more popular, due the greater awareness of consumers to the negative effects of excess dietary intake of sodium. High daily intake of sodium can potentially lead to hypertension and other cardiovascular diseases [16,17]. Reducing the salt content of shrimp paste or fish sauce may also improve its nutritional properties, such as an increase in amino and fatty acids [11,18]. Moreover, using high salt concentration in shrimp paste production could prolong fermentation time. Traditionally, shrimp paste that is naturally fermented using 25–30% salt concentration needs 6 to 12 months of fermentation time [11]. Therefore, a reduced salt content in the processing of fermented food could shorten the fermentation time of fish sauce [18]. Although the use of less salt in terasi is favorable for dietary and economic reasons, it poses health risks, such as the growth of pathogenic bacteria from raw materials and increased proliferation of spoilage bacteria.

In general, bacteria and archaea are usually found in salty fermented food [19,20], with bacteria suggested to be involved in the process of degrading shrimp components into various compounds, such as glutamic acid [1,6], Maillard reaction products [11,21], aspartic acid [6,22], and 5’-nucleotide [1], that contribute to the quality, taste, and flavor of the fermented food [5,20,22]. The characteristics of shrimp paste depend on the period of fermentation, raw material, fermentation process, and salt content [1,5]. Among these, salt content and fermentation period could influence the diversity and enzyme activity of bacteria in shrimp sauce or paste [9,20].

Dynamic changes in the population of bacteria occur during the fermentation of shrimp. During the early fermentation of shrimp sauce or paste, pathogenic bacteria, such as *Vibrio*, *Photobacterium*, *Psychrobacter*, *Pseudoalteromonas*, and *Enterovibrio*, are dominant; however, their population decreased at the end of fermentation [20,23], while the spoilage bacteria *Halanaerobium* increased. In terms of metabolites, an increase in amino acids occurs at the end of shrimp sauce fermentation, along with acetic acid, butyric acid, and trimethylamine, due to the proliferation of spoilage bacteria [20]. Moreover, bacteria in shrimp paste have several enzymes, such as proteases [1,3,10,13,22] and aminopeptidases [9], that could produce free amino acids, as well as lipases that produce fatty acid [13,20]. Enzymatic activity of bacteria in shrimp paste could also produce biogenic amines [10,24]. With these dynamic changes in shrimp paste, the determination of the bacterial population and metabolome profile in shrimp paste is deemed necessary to assess the safety and quality of the fermented product.

To date, there is still no investigation of the microbial diversity and metabolome profile in low-salt shrimp paste during fermentation. Thus, in this study, the microbial succession in low-salt shrimp paste was analyzed using the high-throughput sequencing approach, while the metabolite changes during fermentation were investigated using the untargeted metabolomics approach by GC/MS, in order to understand the microorganisms involved, as well as the corresponding metabolite changes in the shrimp paste fermentation process. In this study, determining the dynamic changes of bacterial population and metabolites in low-salt shrimp paste can provide data on the best time to stop fermentation to obtain a better quality of low-salt shrimp paste.

## 2. Results

### 2.1. Protein Content of Shrimp Paste with Various Salt Concentrations

To know the level of protein content in low-salt shrimp paste, the comparison between shrimp paste with various salt content was done. Based on our comparative analysis of shrimp paste with varying salt concentrations (Table 1), low-salt shrimp paste (5%) showed the highest protein content, making it the ideal salt concentration for producers, in terms of nutritional value.

### 2.2. Changes in Physicochemical Properties and Culturable Bacterial Population

The changes in the physicochemical properties and culturable bacterial population in the shrimp material and low-salt shrimp paste are shown in Table 2. In terms of pH, the shrimp material had higher pH than low-salt shrimp paste, with T0 having the lowest pH at 7.68. On the other hand, the shrimp material at T4 had the highest salinity (7.91%), while T2 had the lowest salinity (6.24%). Additionally, the total culturable aerobic bacteria present in low-salt shrimp paste were lower, compared to the shrimp material. Overall, the total number of culturable aerobic, halophilic, and lactic acid bacteria decreased during fermentation. However, the total halophilic and lactic acid bacteria were higher than aerobic bacteria in low-salt shrimp paste.

### 2.3. Bacterial Communities in Shrimp and Low-Salt Shrimp Paste

To monitor the dynamic changes in microbial population during the low-salt shrimp paste fermentation, the bacterial community analysis of time course samples, from 0 to 28 days of fermentation (T0-T4), was conducted. The total raw reads obtained were 2,267,198, which is divided into 318,816 shrimp, 392,168 T0, 372,690 T1, 400,406 T2, 427,402 T3, and 355,716 T4 reads. A good sequencing result was achieved with >50% GC content, Q20 and Q30 values of >90%, and 0.99 coverage (Table 3). The lowest read count was in shrimp material at 1.6 × 10^5^, while the highest read count was at 2.0 × 10^5^ in T2. The operational taxonomy unit (OTU) obtained in this study ranged from 24 to 114. Chao1 showed that sample T1 (7 days of fermentation) had the highest community diversity. The Shannon and inverse Simpson indexes were used to measure species diversity of the community. The high Shannon index and inverse Simpson value in sample T3 (21 days of fermentation) indicated that this time point had the highest community diversity. In contrast, the shrimp material had the lowest community diversity, as indicated by the low inverse Simpson value (0.38). Thus, the fermentation of shrimp to low-salt shrimp paste resulted in an increase in the diversity of bacterial community, up until 21 days of fermentation, after which, it decreased at the end of fermentation (Table 3).

**Figure 1:**
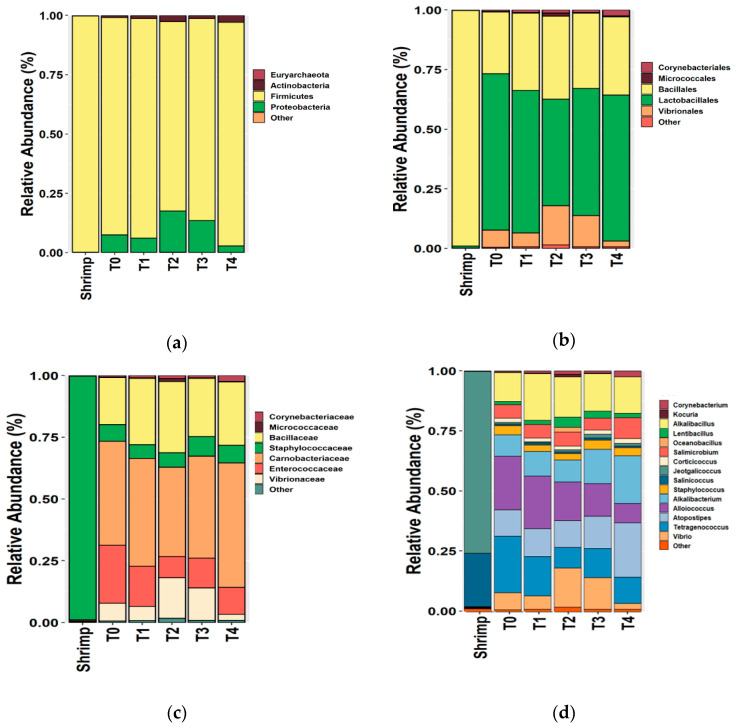
The relative abundance of bacterial OTUs from shrimp material and low-salt shrimp paste at the phylum (**a**), order (**b**), family (**c**), and genus (**d**) levels, revealed by Illumina sequencing, using the V3–V4 variable region of 16S rRNA, and analyzed by QIIME. Only dominant orders, families, and genus were selected (relative abundance more than 1%). T0 = 0 days of fermentation, T1 = 7 days of fermentation, T2 = 14 days of fermentation, T3 = 21 days of fermentation, T4 = 28 days of fermentation.

In terms of bacterial diversity, we revealed the presence of 13 phyla, 23 classes, 39 order, 64 families, 109 genera, and 152 species in the low-salt shrimp paste. In comparison, 3 phyla, 4 classes, 6 order, 12 families, 18 genera, and 22 species were present in the shrimp material. We observed three major phyla (Firmicutes, Proteobacteria, Actinobacteria) in both shrimp material and low-salt shrimp paste, with Firmicutes being the dominant phylum in the latter (Figure 1A). The dominant order of bacteria in the fermentation of low-salt shrimp paste and material were Lactobacillales and Bacillales, respectively (Figure 1B). This result indicated that lactic acid bacteria (LAB) played a significant role in the fermentation process of low-salt shrimp paste. Carnobacteriaceae, Enterococcaceae, and Bacillaceae were the abundant family during the fermentation of low-salt shrimp paste, at 36.23–50.44%, 8.47–23.46%, and 19.10–28.87%, respectively.

**Figure 2:**
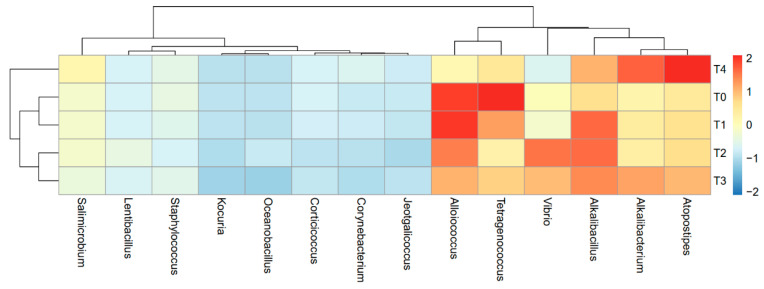
Heatmap showing the relative abundance of dominant bacterial genus in the low-salt shrimp paste, using Illumina sequencing of the V3–V4 variable regions of 16S rRNA.

This study showed a dynamic change in the dominant bacterial population during the fermentation of low-salt shrimp paste (Figure 2 and Table S1). At the early stage of fermentation (T0), *Tetragenococcus* and *Alloiococcus* were predominant. In contrast, *Atopostipes* and *Alkalibacterium* were the dominant bacteria at the last stage of fermentation (28 days). As shown in the heatmap dendrogram, the T0 and T1 and T2 and T3 time points showed similarity, in terms of the dominant bacteria, while T4 contained the most diverse bacterial composition among the time points.

### 2.4. Detection of Pathogenic Bacteria (Escherichia coli and Salmonella) Culture-Dependent Method

The results showed that, during fermentation, enteropathogenic bacteria, such as *E. coli* and *Salmonella*, were not detectable in the eosin-methylene blue agar (EMBA) medium for *E. coli*, salmonella-shigella agar (SSA), and xylose lysine deoxycholate agar (XLD agar) for *Salmonella* (Table 4). All of the samples were enriched before growing in selective media for growth of pathogenic bacteria using lactose broth and endo broth for *E. coli*, lactose broth and tetrathionate broth for *Salmonella.* This implies that low-salt shrimp paste was safe to consume.

### 2.5. Metabolite Composition of Low-Salt Terasi

**Figure 3:**
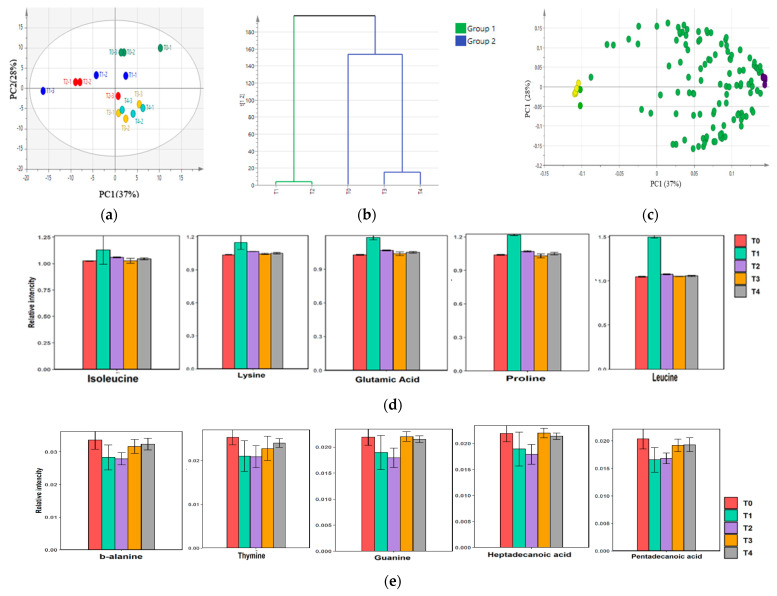
Principle component analysis (PCA) score plot and loading plot of low–salt shrimp paste at different time points during fermentation. Data were acquired from GC/MS analysis of triplicate samples, using ribitol as standard and multivariate analysis, using SIMCA 13.0; (**a**) score plot, (**b**) hierarchical cluster analysis, (**c**) loading plot; yellow dots = marker for T1 and T2; purple dots = marker for T0, T3, and T4. (**d**) Top five scores of eigenvector value metabolites correlated with T1 and T2. (**e**) Five top scores of eigenvector value metabolites correlated with T0, T3, and T4. T1 = 7 days of fermentation, T2 = 14 days of fermentation, T3 = 21 days of fermentation, T4 = 28 days of fermentation.

Metabolite profiling by GC/MS produced 121 annotated metabolites, as shown in Table S2. Specifically, 22 sugars (mono and disaccharides, sugar alcohols, purine, and pyrimidine derivatives), 24 carboxylic acids, 43 amino acids, 15 fatty acids, 9 amines/amides, 5 phenolics, and 3 other metabolites were identified. To monitor the metabolite changes during shrimp paste fermentation, metabolite analysis was conducted using time course sampling from day 0 to day 28 (T0–T4). PCA analysis was conducted using the 121 metabolites as the explanatory variable. Changes in the metabolome profile were observed during the duration of shrimp fermentation, as shown in Figure 3.

**Figure 4:**
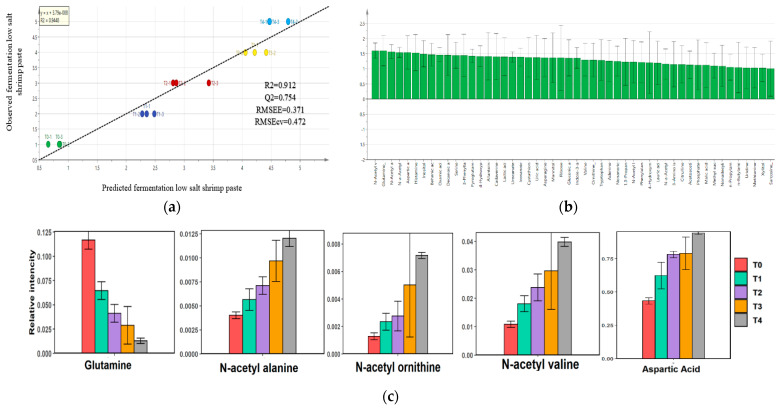
Predicted model of low-salt shrimp paste fermentation. (**a**) OPLS model, (**b**) VIP of metabolites during fermentation of low-salt shrimp paste, and (**c**) top five scores of VIP metabolites during fermentation of low-salt shrimp paste.

The PCA loading plot was examined to determine the metabolite changes from beginning to completion of low-salt shrimp paste fermentation. Hierarchical clustering analysis, based on PCA, of the mean value of each metabolite in low-salt shrimp paste showed two clusters, T1 and T2 in one group and T0, T3, and T4 in another group. The loading plot indicated that amino acids, such as isoleucine, lysine, glutamic acid, proline, and N-acetyl glucosamine, contributed to the separation of T1 and T2, while purine/pyrimidine and fatty acids, such as β-alanine, thymine, guanine, heptadecanoic acid, and pentadecanoic acid, contributed to the separation of T0, T3, and T4. Next, the OPLS model was constructed to determine important metabolites correlated to the maturity of low-salt shrimp paste. VIP from OPLS showed that, from day 0 to day 28 of fermentation, glutamine levels dropped, while N-acetylated amino acids (N-acetyl valine, N-acetyl alanine, and N-acetyl ornithine) and aspartic acid increased (Figure 4).

**Figure 5:**
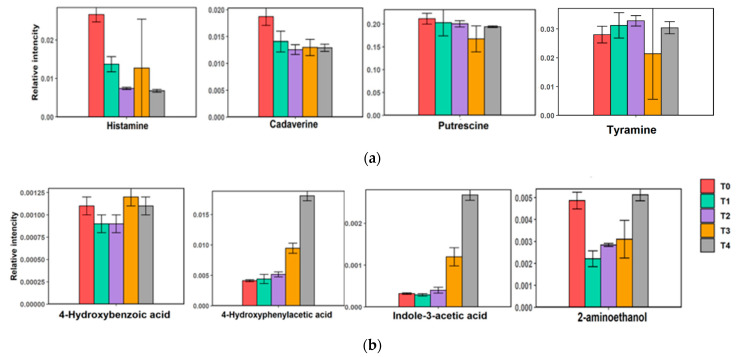
Biogenic amines production and decomposition of amino acids during fermentation of low-salt shrimp paste. (**a**) Biogenic amines; (**b**) decomposition of amino acids compounds during fermentation of low-salt shrimp paste.

Detection of biogenic amines in the sample is an indication of amino acids decomposition. In our results, high biogenic amine levels occurred at day 0 of low-salt shrimp paste fermentation (Figure 5); however, as fermentation progressed, levels of amine compounds, such as cadaverine and histamine, decreased. Further decomposition of amino acid products (putrefaction), such as indole-3-acetic acid and phenol derivatives (4-hydroxyphenylacetic acid), increased after 21 days of fermentation (Figure 5).

**Figure 6:**
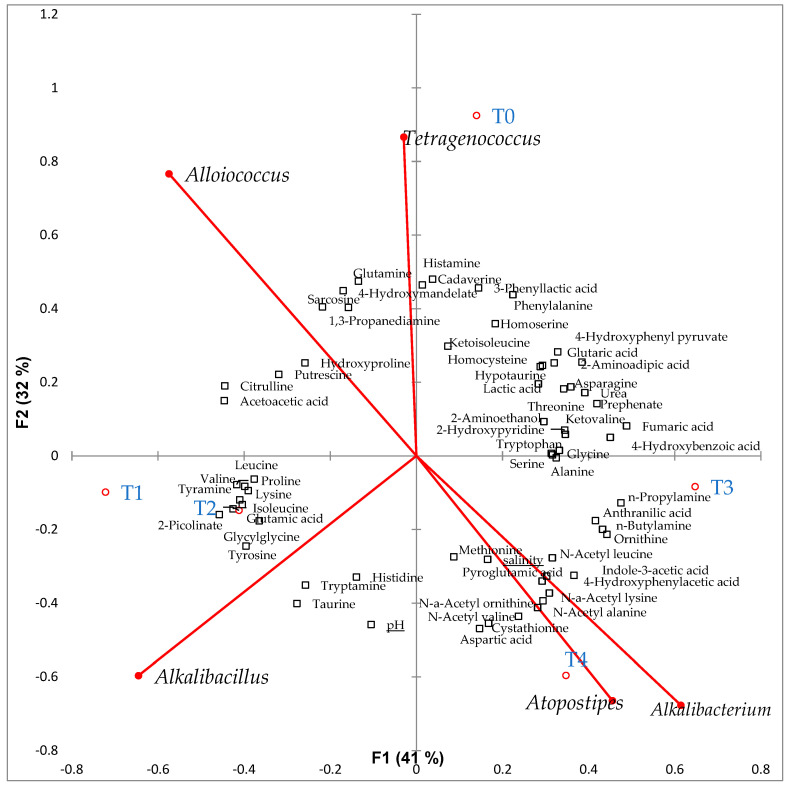
Redundancy analysis (RDA) showing the relationship between some metabolites (amino acids, amino acid’s degradation products, biogenic amines, and some putrefaction compounds) and dominant bacteria in low–salt shrimp paste during fermentation. Black square = metabolites, pH, and salinity; red dot = low–salt shrimp paste; red line = bacteria. RDA were analyzed using XL–STAT.

To show the relationship between metabolites (amino acids, biogenic amines, and some compounds) and the dominant bacteria in low-salt terasi during fermentation, RDA analysis, using XL-STAT, was conducted. *Tetragenococcus* correlated with histamine and cadaverine (*p* < 0.05), while *Atopostipes* correlated with indole-3-acetic acid and 4-hydroxyphenylacetic acid (*p* < 0.05) (Figure 6).

## 3. Discussion

The low total viable count of culturable aerobic bacteria in low-salt shrimp paste, compared to halophilic and lactic acid bacteria, indicated that the growth of putrefaction or spoilage bacteria was suppressed by halophilic or lactic acid bacteria (LAB). Halophilic bacteria play a vital role in protein hydrolysis and the formation of the unique flavor and aroma of shrimp paste [25]. Lactic acid bacteria, in particular, play a role in flavor formation in fermentation products, due to their ability to ferment carbohydrates [26]. The pH was lowest at T0, due to the accumulation of acid products. In Chinese shrimp paste fermentation, the decrease in pH was reportedly due to the accumulation of acid produced by lactic acid bacteria [25]. In this study, lactic acid bacteria predominated at T0, as indicated by the high counts on MRS agar, resulting in the lowering of pH. The increase in pH at T1, until T4 of the fermentation period, might be caused by the formation of degradation products or volatile basic compounds, such as ammonia [10,15,25]. The aerobic bacteria present in the shrimp material were higher than in low-salt shrimp paste. Salting and drying procedures in the shrimp paste production process could reduce the moisture content and may contribute to the inhibition of growth of putrefaction or spoilage bacteria in shrimp paste [15].

In this study, OTU and bacterial diversity indices were lower in the shrimp material than in low-salt shrimp paste. Furthermore, we found that bacterial diversity tends to increase during fermentation, until the 21st day (T3). Afterwards, a decrease in bacterial population was observed at the end of fermentation. A similar result was observed after comparing the shrimp material with kapi (Thailand’s shrimp paste), where increased diversity index was found in kapi after one month of fermentation. This was followed by a decreasing trend in the diversity index, until two months of fermentation [9]. Similar to kapi, the increase in bacterial diversity may be due to the high nutritional content during shrimp fermentation, as a result of the degradation of proteins, chitin, and glycogen to amino acids and monosaccharides [15]. The decrease in bacterial diversity in low-salt shrimp paste at the end of fermentation (28 days) may be caused by fermentation products that suppress bacterial growth, such as p-cresol and phenol [26]. In particular, p-cresol is a result of the degradation of tyrosine and phenylalanine via 4-hydroxyphenylacetic acid [27,28]. In this study, 4-hydroxybenzoic acid (phenol derivative of benzoic acid) was abundant at T3 and T4, while 4-hydroxyphenylacetic acid sharply increased at T4.

The phylum composition of low-salt shrimp paste showed similarity with the Chinese fermented shrimp paste. The dominance of Firmicutes in low-salt terasi is in line with other studies, such as in Chinese [2,23,25] and Thai shrimp paste (kapi) [9]. At the end of fermentation (28 days), a reduction in Proteobacteria was observed. The presence of Proteobacteria is typically indicative of the freshness and hygiene conditions of shrimp paste [2,23]. In Huanghua and Jinzhou shrimp pastes, a higher relative abundance of Enterococcaceae was observed [2,25]. In low-salt shrimp paste, we found that the dominant genera were *Alkalibacillus*, *Alkalibacterium*, *Alloicoccus*, *Atopostipes*, *Tetragenococcus*, *Vibrio*, and *Salimicrobium*. In contrast, the Chinese shrimp paste was dominated by *Tetragenococcus, Pseudomonas, Staphylococcus, Lactobacillus, Psychrobacter, Halomonas, Bacillus, Alteribacillus,* and *Lactococcus* [2,23,25], while *Lentibacillus, Salinococcus, Salimicrobium, Alkalibacterium, Staphylococcus, Jeotgalicoccus psychrophilus*, and *Bacillus* were the dominant genera in Thailand shrimp paste [9].

The shrimp material used in this study, *Acetes japonicus*, was different from the planktonic shrimp (*Acetes* spp.) used as raw material for the Thai shrimp paste (kapi). Thus, it might have contributed to the difference in the most common bacterial genera found in this study (*Jeotgalicoccus*), as well as the one found in kapi (*Vagococcus* spp.) [9]. In addition, the differences in bacterial composition reported in this study, compared to previous studies on other types of fermented shrimp from different countries, may be due to variation in the salt concentration used, length of fermentation, and source of raw materials. In this study, we used a lower salt concentration (salinity = 6.24–7.91%), compared to other shrimp pastes from Thailand (salinity = 15.36–34.43%) and China (salinity = 20.78–21.26%) [9,25].

At T0, T3, and T4, the levels of purine and pyrimidine compounds, such as guanine, thymine, β-alanine (degradation product of thymine), and 15–17 carbon chain fatty acids, were high. At T0, purine and pyrimidine compounds were probably derived from the degradation of shrimp nucleotides, while at T3 and T4, purine and pyrimidine compounds came from bacterial nucleotides. Moreover, at the start of fermentation (T0), 15–17 carbon chain fatty acids presumably originated from the lipid degradation of shrimp. In fact, lipid degradation and oxidation products, such as aldehydes [25], peroxide value (PV), and thiobarbituric acid reactive substances (TBARS) were detected during shrimp paste fermentation [15]. The degradation of shrimp lipoprotein to free astaxanthin was also observed during fermentation. Astaxanthin gives the red color of shrimp paste (that can be detected as a* value) and is known as an antioxidant [3,11]. At T3, the highest a* value was obtained, indicating that the shrimp paste had a more intense red color and high free astaxanthin levels.

At the initial stage of fermentation (T0), amino acids in shrimp were degraded, which can be detected as biogenic amines at T0. Additionally, some of the amino acids previously synthesized from the protein degradation have been degraded later at T3 and T4. Putrefaction products, such as indole-3-acetic acid and 4-hydroxyphenylacetic acid, that are low in amount at the early stages of fermentation were found to be elevated after 21 days of fermentation. In contrast, histamine and cadaverine have the tendency to decrease during the fermentation process. Bacteria found in fermented food have enzymes, such as histamine oxidase or histamine dehydrogenase, that can degrade histamine to imidazole acetaldehyde and ammonia [29]. In addition, cadaverine can also be broken down into glutaric acid [30]. Although a species from *Tetragenococus*, such as *T. muriaticus*, plays an important role in the fermentation of food [31,32,33,34], it is also known to produce histamine [35,36] and cadaverine [37], as previously reported in Huanghua shrimp paste [8].

A good model of OPLS has an R2 value of more than 0.6, Q2 value of more than 0.6, and a low value of RMSEE and RMSECV [38,39]. Based on these criteria, the OPLS model of low-salt shrimp paste in this study had a good fit. During the fermentation of low-salt shrimp paste, glutamine levels dropped, which was also observed in the fermentation saeu-jeout or small shrimp [19]. Glutamine is an unstable amino acid and ammonia source [40,41]. Acetylated amino acids increase during fermentation and can change the food’s sensory characteristics, resulting in sour taste or tastelessness [42]. Generally, acetylation of amino acids depends on two significant factors: rapid carbon flux and a carbon-nutrient imbalance that restricts the growth of bacteria [43,44]. At some point, acetylation occurs as bacteria enter the stationary phase of growth [44]. Community abundance, based on Chao level and bacterial count, has a tendency to decrease at the end of fermentation, which indicated that some bacteria entered the death phase of growth.

Species of genus *Atospostipes*, such as *Atopostipes suicloacalis*, is a heterofermentative lactic acid bacterium usually found in swine manure pit that is rich in ammonia, phenol, indole, benzoic acid, alcohol, and volatile fatty acids (acetic acid, isobutyric acid, and isovaleric acid) [45,46,47,48]. In this study, there was a correlation between *Atopostipes* and indole-3-acetic acid and 4-hydroxyphenylacetic acid. On the other hand, it is assumed that the decomposition of protein and amino acids in low-salt terasi created a nitrogen-rich environment, suitable for the growth of *Alkalibacterium*, such as *Alkalibacterium putridalgicola,* a marine lactic acid bacterium [49], usually found in nitrogen-enriched environment during the decay of seaweed [50].

Taste precursors in shrimp paste are determined by Maillard reaction products [5,11] and umami-enhancing amino acids, such as glutamic [5] and aspartic acid (sweet taste) [4,51]. Maillard reaction products can be detected by the yellow color (indicated as b* value) in the CIELAB colorimetric test [22,52]. The b* value is a measure of the yellowness of food. In kapi, an increase in b* indicated that lipid oxidation products, such as the carbonyl group, reacted with amino group to produce a Maillard reaction, leading to the formation of yellow color [3]. In this study, T3 stage had the highest b* value, indicating that Maillard reaction products accumulated on day 21 of fermentation. In this study, levels of glutamic acid and essential amino acids (leucine, isoleucine, and lysine) were highest at T1. Aspartic acid increased until the end of fermentation.

Low-salt shrimp paste fermentation can be a good alternative method for processing of planktonic shrimp to shrimp paste. Based on our findings, low-salt shrimp paste has high protein content, compared to high-salt shrimp paste. Using low-salt concentration can also shorten the fermentation time to 1–3 weeks. The use of low-salt in shrimp paste fermentation is beneficial, as it can produce a stable product that is less likely to be contaminated with pathogenic bacteria, such as *E. coli* and *Salmonella*. However, it is necessary to examine the shelf life of low-salt shrimp paste, quench bacterial enzymatic activity, and apply preservation techniques, such as vacuums and hot or cold temperature treatment cells, before consumption. It was reported that heat treatment of fermented shrimp using 12.5% of salt could be employed to increase stability and enhance the antioxidant property of shrimp paste [53]. As previously reported in kimchi preservation, high pressure CO_2_ treatment may be used as a non-thermal technique to quench bacterial enzymatic activity that degrades nutrients and may be used in low-salt shrimp paste production [54].

The findings of this study will be helpful in the fermented food industry, in order to assess the optimal length of fermentation time for the production of good quality shrimp paste or terasi. We also found that high levels of glutamic and essential amino acids are present at the T1 and T2 stages (7 and 14 days), and there are increased Maillard reaction products at the T3 stage (21 days); it is recommended that the fermentation should be stopped on the 7th day and should not go over 21 days. At the T4 stage of fermentation (28 days), putrefaction products, such as indole-3-acetic acid and 4-hydroxyphenylacetic acid, have already accumulated. The heatmap showed that the bacterial community at the T4 stage was most different, compared to the other stages of shrimp paste fermentation.

## 4. Materials and Methods

### 4.1. Preparation of Shrimp Paste (Terasi)

In this study, fermentation was conducted twice. Semi-dried shrimp (*Acetes japonicus*) was mixed with solar salt to make low-salt shrimp paste with a 5% salt concentration. Additionally, shrimp paste with 10%, 15%, and 20% salt concentrations were prepared using various amounts of solar salt. After grinding, the first fermentation was performed for two days (48 h) to process shrimp into low-salt shrimp paste with varying salt concentration. After sun-drying, the materials were ground, and 1 g was taken for protein content measurement. After grounding, low-salt shrimp paste was allowed to ferment further (second fermentation), for 28 days (4 weeks), leading to the maturation of shrimp paste. Time course sampling was done once a week, during the duration of the second fermentation. Sampling at days 0, 7, 14, 21, and 28 of the second fermentation were designated as T0, T1, T2, T3, and T4, respectively. Ten grams each was taken from both the shrimp and low-salt *terasi* for analysis of bacterial communities and metabolites.

### 4.2. Protein Content of Shrimp Paste

Analysis of protein concentration was tested by measuring the nitrogen content in the food ingredients. Protein concentration was measured using a Foss Tecator Kjeltec 8400 [55]. One gram of shrimp paste was inserted into the Kjeldahl flask and mixed with 2 g of a mixture of selenium (2.5 g Selenium, 100 g of K_2_SO_4_, 20 g of CuSO_4_.5H_2_O) and 25 mL concentrated H_2_SO_4_, before being heated over an electric heater or burner fire. The mixture was boiled until it becomes clear and greenish. This mixture was allowed to cool before dilution and transfer into a 100 mL volumetric flask. Five mL of the solution was pipetted and put into a distiller. Five millilitres of 30% NaOH was added in the solution with a few drops of phenolphthalein indicator (PP). Distillation was performed for over 10 min, with 10 mL of 2% boric acid solution mixed with the indicators as the reservoir. The distilled result was transferred into an Erlenmeyer before being titrated with 0.01 N HCl solution. Calculation of protein concentration was performed using the following formula:

$$\begin{array}{l} \%\;\mathrm{Nitrogen}=\frac{\mathrm{Vol}.\;\mathrm{HCl}\;\left(\mathrm{mL}\right)-\mathrm{Vol}.\;\mathrm{blanko}\left(\mathrm{mL}\right)-N\;\mathrm{HCl}\times 14.007}{\mathrm{weight}\;\mathrm{sample}\;\left(\mathrm{mg}\right)}\\ \%\;\mathrm{protein}\;\mathrm{content}\;=\;\%\;\mathrm{Nitrogen}\;\times 6.25 \end{array}$$

### 4.3. Physicochemical Analysis and Enumeration of Bacteria

The pH of the sample was measured using a pH meter (Ohaus ST 2100-F, USA). The salt and moisture content were analyzed using the Mohr and thermogravimetry methods, respectively [56]. The enumeration of culturable total bacteria was performed by total viable count on selective media, while plate count agar was used to enumerate the aerobic bacteria [11]. For lactic acid and halophilic bacteria, de Man–Rogosa–Sharp agar, supplemented with 5% NaCl [10] and salt marine agar, were used, respectively [57]. Plate count agar, salt marine agar, de Man–Rogosa–Sharp agar for cultural bacteria were purchased from Hi-Media (India). All samples were subjected to CIELAB colorimetric L* (light-ness), a* (redness-greenness), and b* (yellowness-blueness) tests, using Konica Minolta (11).

### 4.4. Detection of Pathogenic Bacteria (E. coli and Salmonella) in Low-Salt Shrimp Paste Qualitatively Used Culture-Dependent Method

Detection of *Escherichia coli* was achieved according to [58] with modification. A total of 25 g of shrimp paste was placed in a sterile plastic bag with 225 mL Butterfield phosphate buffer (BPB), homogenized using vortex (Thermoscientific, Waltham, MA, USA) for 2 min. A tenfold serial dilution was carried out, and 1 mL of dilution 10^−1^, 10^−2^, and 10^−3^ were transferred to lactose broth medium (LB), with a Durham tube inside, and incubated at 35 ± 1 °C for 24–48 h in an incubator (Memmert, Schwabach, Germany). After incubation, each LB tube showing gas formation was transferred to Endo broth medium. From the Endo broth media showing positive results, a loopful was taken and streaked on the eosin-methylene blue agar (EMBA) medium. Flat colonies with dark centers, with or without metallic sheen on EMBA medium, were tentatively identified as *E. coli* for further confirmation and streaked on PCA slant. IMVIC tests (indole, methyl red, Voges-Proskauer, and Simon citrate) and Gram staining were performed to confirm the results.

Detection of *Salmonella* was achieved according to [58], with modification. A total of 25 g of shrimp paste was put into a sterile plastic bag, with 225 mL of lactose broth, homogenized for 2 min, transferred to a sterile Erlenmeyer flask, and left at room temperature for 60 min. Before being incubated at 35 ± 1 °C for 24 ± 2 h, the Erlenmeyer flask was gently shaken and then covered with a cotton lid. After 24 h incubation, 1 mL of incubated LB was transferred to 10 mL of tetrathionate broth medium (TTB) and incubated at 35 ± 1 °C for 24 ± 2 h. Then, a 3 mm loopful of incubated TTB was streaked on salmonella-shigella agar (SSA) and xylose lysine deoxycholate agar (XLD agar) and incubated at 35 ± 1 °C for 24 ± 2 h. Positive results were indicated by transparent colonies with a black center on SSA and pink colonies with a black center on XLD agar. For further tests, slant agar of triple sugar iron agar (TSIA) and lysine iron agar (LIA) were used by stabbing the bottom of the tube and streaking the slant media using an inoculum needle. Positive results were indicated by the yellow color at the bottom of the tube, from both on TSIA medium and LIA medium, while brick-red slant on the TSIA and purple slant on LIA.

### 4.5. Bacterial Communities Analysis in Shrimp and Shrimp Paste

#### 4.5.1. Extraction of DNA

Extraction of DNA was done using the DNA miniprep kits (Zymoresearch Catalog 4300), following the manufacturer’s instructions. Briefly, 300 µg of the low-salt terasi were placed into a lysis tube containing lysis buffer. Proteinase K (Zymoresearch, Irvine, CA, USA) (10 µL) was added, and the mixture was incubated at 55 °C for 60 min. The PicoGreen method, using the Victor 3 fluorometer, was used for quantifying the metagenomic DNA.

#### 4.5.2. PCR Amplification and Sequencing

The extracted metagenomic DNA was used as template for PCR amplification, following the method described in Qubit 2.0 (Life United States). Two universal primers, forward 341F (CCTACGGGNGGCWGCAG) and reverse 805R (GACTACHVGGGTATCTAATCC), were used to amplify the V3–V4 regions of bacterial 16S rRNA. The primer set contained the appropriate Illumina adapters, with the reverse primer containing a unique, error-correcting barcode for each sample. The PCR reaction included two rounds of amplification. In the first round of amplification, the PCR mixture contained 15 μL of 2× Taq Master Mix, 1 μL of PCR primer F (10 uM), 1 μL of primer R (10 uM), 20 ng of genomic DNA, and ultra-pure H_2_O to give a final reaction volume of 30 μL. PCR amplification was performed using a T100^TM^ Thermal Cycler (BIO-RAD, Berkeley, CA, USA). The first program was as follows: 1 cycle of denaturation at 94 °C for 3 min, 5 cycles of denaturation at 94 °C for 30 s, annealing at 45 °C for 20 s, elongation at 65 °C for 30 s, then 20 cycles of denaturation at 94 °C for 20 s, annealing at 55 °C for 20 s, elongation at 72 °C for 30 s, and a final extension at 72 °C for 5 min. In the second round of amplification, the PCR mixture contained 15 μL of 2× Taq master mix, 1 μL of primer F (10 uM), 1 μL of primer R (10 uM), 20 ng of genomic DNA, and ultra-pure H_2_O to give a final reaction volume of 30 μL. PCR amplification of the 16S rRNA V3–V4 regions was performed using a T100^TM^ Thermal Cycler. The second amplification program was as follows: 1 cycle of denaturation at 95 °C for 3 min, then 5 cycles of denaturation at 94 °C for 20 s, annealing at 55 °C for 20 s, elongation at 72 °C for 30 s, and a final extension at 72 °C for 5 min. The PCR products were checked by electrophoresis using 1% (*w*/*v*) agarose gel in TBE (Tris, boric acid, EDTA) buffer and stained with nuclease dye. The PCR products were visualized under UV light. Agencourt AMPure XP beads (Beckman, Indianapolis, IN, USA) were used to purify the free primers and primer–dimer species in the amplification product. Before sequencing, the DNA concentration of each PCR product was determined using a Qubit 2.0 kit, and its quality was controlled using a bioanalyzer (Agilent, Santa Clara, CA, USA) [23], with modification.

Following the manufacturer’s recommendations, a sequencing library was prepared using Herculase II Fusion DNA Polymerase Nextera XT Index Kit V2 (Agilent Technologies, Inc., Santa Clara, CA, USA). Library quality was assessed by Qubit 2.0 fluorometer (Thermo Scientific, Waltham, MA, USA) and Agilent Bioanalyzer 2100 system using a DNA 1000 chip. Prepared libraries were quantified using qPCR according to the Illumina qPCR Quantification Protocol Guide. According to the platform’s instructions, the library was sequenced on an Illumina MiSeq platform (Illumina, San Diego, CA, USA). The sequencing was done by Macrogen Inc., Korea, following the company’s standard procedure.

#### 4.5.3. Data Analysis

Data analysis consisted of three steps: pre-processing and clustering, taxonomy assignment, and diversity statistics. Ambiguous reads were filtered out, and the extra-long tail was trimmed. Short reads were assembled using the FLASH (1.2.11) program (http://ccb.jhu.edu/sofware/FLASH/) accessed on 3 May 2019. Pre-processing and clustering using CD-HIT-OTU (http://weizhongli-lab.org/cd-hit-otu/) accessed on 7 May 2019. Chimeras were identified and removed using rDNA tools PacBio (https://github.com/PacificBiosciences/rDnaTool) accessed on 7 May 2019 and reference file (RDP) http://www.mothur.org/wiki/RDP_reference_file accessed on 23 May 2019. QIIME-UCLUT/RDP and QIIME were used for taxonomy assignment (16S) and diversity statistics analysis (OTUs, Chao1, Shannon, and inverse Simpson), respectively. Two strategies for OTU’s picking were used de novo and open reference using RDP database. Taxonomy assignment also compared to SILVA 123 reference. The bar graphs used for taxonomy and heatmap representation of dominant bacteria (relative abundance more than 1%) were performed using R package ggplot2 and pheatmap.

### 4.6. Metabolites Detection in Shrimp Paste

#### 4.6.1. Preparation of Samples

The low-salt terasi samples were lyophilized using a freeze-drying machine. Samples were crushed using a multi-bead shocker. The compounds were extracted from low-salt terasi samples (10 mg) using 1 mL of 80% methanol (Wako Pure Chemical Industries, Osaka, Japan) with 0.2 mg/mL ribitol (Wako Pure Chemical Industries, Osaka, Japan) as an internal standard. The mixture was incubated in a shaker incubator at 161× *g* for 20 min at 37 °C. After incubation, the mixture was centrifuged at 11,740× *g* for 10 min at 4 °C. After mixing with vortex, the mixture was centrifuged at 11,740× *g* for 3 min at 4 °C. Next, 200 µL of the supernatant was transferred to a fresh 1.5 mL microfuge tube. The supernatant was centrifuged at 3× *g* for 90 min at 25 °C. The extract was dried under vacuum overnight (10–12 h) and freeze-dried until completely dried. Derivatization was carried out in a shaker incubator at 161× *g* at 30 °C for 90 min, with 100 µL of methoxyamine hydrochloride in pyridine (Sigma Aldrich, Missouri, United States, 20 mg/mL in pyridine). Fifty microliters (50 µL) of 2,2,2-trifluoro-N-methyl-N-trimethylsilylacetamide (GL Sciences, MSFTA) were added, and the mixture was incubated in a shaker incubator at 161× *g* at 37 °C for 30 min. Next, 100 µL of the sample was transferred into GC/MS vial.

#### 4.6.2. GC/MS Analysis

GC/MS analysis was carried out using GC-Q/MS; GCMS-QP 2010 Ultra (Shimadzu, Kyoto, Japan) was installed with an Inertcap 5 MS/NP column (30 m × 0.25 mm I.D., df. = 0.25 µm) (Variant Inc., Palo Alto, CA, USA). Beforehand, the MS analysis was adjusted and calibrated. The sample aliquot, amounting to 1 µL, was injected in split mode, 25/1 (*v*/*v*), with an injection temperature of 230 °C. Carrier gas flow (HELIUM) was 1.12 mL/min, with a 39 cm/sec linear velocity. The column temperature of 80 °C for 2 min was elevated every 15 min to reach 330 °C for 6 min. The transfer line temperature and ion source were set to 250 °C and 200 °C, respectively. Electron ionization (EI) of 0.93 kV was set to produce ions, and the spectrum was recorded by 10,000 u/sec, with a mass range of 85–500 *m*/*z.*

#### 4.6.3. Data Processing

Chromatographic data from GC/MS were pre-processed using MS-DIAL to detect peaks, initial corrections, and alignment of retention times. The spectrum was normalized manually by adjusting each sample’s peak intensity with internal standards (ribitol). The multivariate statistical analyses on metabolites were performed with the SIMCA-13.0 software (Umetricus AB, Sweden). Principal component analysis (PCA) was applied for each metabolite in triplicate. PCA was performed, in order to know the differences among the samples during fermentation. Hierarchical cluster analysis (HCA) was performed based on the mean value of each sample to observe the distance amongst observations, which was calculated using Ward’s method [59]. Orthogonal projections to latent structures (OPLS) regression model was used to reduce the variables [38] and predict metabolites, in terms of the maturity of low-salt shrimp paste. From OPLS analyses, variable importance in projection (VIP) was calculated for each metabolite [39]. The bar graphs used to represent the metabolites were created using R package ggplot2.

## 5. Conclusions

Low-salt is a good technique to preservative planktonic shrimp to be shrimp paste, instead high-salt. In terms of the microbiome profile, low-salt shrimp paste (terasi) was dominated by lactic acid bacteria (LAB). To produce good quality of shrimp paste, the fermentation of low-salt shrimp paste can be stopped at seven days or prolonged, but it should not exceed 21 days. After fermentation, the low-salt shrimp paste can be applied with other preservation techniques to stop bacterial enzymatic activity and over fermentation to produce a stable product for industrial application. Therefore, the use of low-salt in shrimp paste production can be advantageous, as it limits the growth of pathogenic bacteria, has higher nutrient content, and has a shorter fermentation time. The findings of this study will be helpful in the fermented food industry, to assess the optimal length of fermentation time for the production of good quality shrimp paste or terasi.

## Figures and Tables

**Table 1:** Protein content of shrimp paste with different salt concentrations, after 48 h of fermentation. Protein content was analyzed via the Kjehdal method in three replicates.

Shrimp Paste	Protein Content *
5% salt	36.03 ± 0.14
10% salt	33.56 ± 0.25
15% salt	31.23 ± 0.30
20% salt	28.89 ± 0.36

* = average of three replicate ± standard deviation.

**Table 2:** Physicochemical and cultural bacteria changes in shrimp material and low-salt shrimp paste during fermentation.

Physicochemical and Culturable Bacteria	Shrimp	T0	T1	T2	T3	T4
pH	8.26 ± 0.01 ^a^	7.68 ± 0.02 ^b^	7.80 ± 0.02 ^ab^	7.81 ± 0.01 ^ab^	7.80 ± 0.04 ^ab^	7.84 ± 0.02 ^ab^
Salinity (%)	3.78 ± 0.25 ^c^	6.65 ± 0.49 ^b^	7.32 ± 0.55 ^ab^	6.24 ± 0.46 ^b^	7.20 ± 0.54 ^ab^	7.91 ± 0.59 ^a^
Moisture (%)	50.46 ± 1.82 ^a^	43.59 ± 0.48 ^b^	44.04 ± 0.56 ^b^	43.28 ± 0.75 ^b^	43.25 ± 1.06 ^b^	43.18 ± 1.67 ^b^
L *	ND	44.05 ± 0.54 ^a^	43.52 ± 1.87 ^a^	45.39 ± 0.54 ^a^	44.54 ± 1.58 ^a^	43.96 ± 1.97 ^a^
a *	ND	5.64 ± 0.21 ^a^	5.90 ± 0.22 ^a^	5.39 ± 0.17 ^a^	6.41 ± 0.60 ^a^	5.39 ± 0.93 ^a^
b *	ND	5.76 ± 0.54 ^b^	5.52 ± 0.76 ^b^	5.51 ± 0.16 ^b^	7.37 ± 0.69 ^a^	6.71 ± 0.54 ^ab^
Total aerobic bacteria (log CFU/g)	5.39 ± 0.22 ^a^	5.25 ± 0.24 ^a^	4.48 ± 0.33 ^b^	4.01 ± 0.12 ^c^	3.73 ± 0.26 ^d^	3.57 ± 0.14 ^d^
Total lactic acid bacteria (log CFU/g)	ND	5.56 ± 0.13 ^a^	5.19 ± 0.55 ^a^	4.96 ± 0.43 ^ab^	4.74 ± 0.29 ^ab^	4.03 ± 0.21 ^b^
Total halophilic bacteria (log CFU/g)	ND	6.43 ± 0.02 ^a^	5.44 ± 0.16 ^b^	5.18 ± 0.24 ^bc^	4.92 ± 0.53 ^c^	4.72 ± 0.25 ^c^

Values were the average of three replicates. Mean with different lowercase superscript, horizontally, was significantly different (one way analysis of variance, *p* < 0.05). The T0 = 0 days of fermentation, T1 = 7 days of fermentation, T2 = 14 days of fermentation, T3 = 21 days of fermentation, T4 = 28 days of fermentation, ND = not determined, CFU = colony forming unit.

**Table 3:** The summary of Illumina sequencing of V3–V4 regions of 16S rRNA, assembled by the FLASH program, showing the quality of the sequencing results (columns 2 to 7). OTUs of metagenome bacteria and diversity index from shrimp and low-salt terasi, analyzed by the QIIME program (columns 8 to 12).

Sample (1)	Total Bases (2)	Read Count (3)	N (%) (4)	GC (%) (5)	Q20 (%) (6)	Q30 (%) (7)	OTU(s) (8)	Chao1 (9)	Shannon (10)	Inverse Simpson (11)	Good Coverage (12)
Shrimp	7.3 × 10^7^	1.6 × 10^5^	0.0001	53.14	99.18	97.22	24	29.00	1.02	0.38	0.99
T0	9.0 × 10^7^	1.9 × 10^5^	0.0001	52.94	99.17	97.15	108	120.05	3.23	0.85	0.99
T1	8.5 × 10^7^	1.8 × 10^5^	0.0001	52.97	99.19	97.19	111	123.75	3.28	0.86	0.99
T2	9.2 × 10^7^	2.0 × 10^5^	0.0001	52.99	99.16	97.12	114	121.56	3.52	0.88	0.99
T3	8.6 × 10^7^	1.8 × 10^5^	0.0001	53.17	99.19	97.20	105	106.50	3.67	0.89	0.99
T4	8.1 × 10^7^	1.7 × 10^5^	0.0001	52.75	99.20	97.22	82	85.11	3.37	0.87	0.99

T0 = 0 days of fermentation, T1 = 7 days of fermentation, T2 = 14 days of fermentation, T3 = 21 days of fermentation, T4 = 28 days of fermentation.

**Table 4:** Detection of pathogenic bacteria (*E. coli* and *Salmonella*) in low-salt shrimp paste, qualitatively used as the culture-dependent method.

Sample	*E. coli* (Presence/Absence in 25 g)	*Salmonella* (Presence/Absence in 25 g)
T0	-	-
T1	-	-
T2	-	-
T3	-	-
T4	-	-

Mark “-“absence in 25 g of samples.

## Data Availability

Data of high-throughput sequencing output in Sequence Read Archive (SRA) in NCBI, with accession PRJNA797953.

## Supplementary Materials

The following supporting information can be downloaded at: https://www.mdpi.com/article/10.3390/metabo12020118/s1. Table S1: Table genus composition of shrimp and low-salt shrimp paste, with abundancy of more than 0.1%, which was revealed by Illumina sequencing, using V3–V4 variable of 16S rRNA. T0 = 0 days of fermentation, T1 = 7 days of fermentation, T2 = 14 days of fermentation, T3 = 21 days of fermentation, T4 = 28 days of fermentation. Table S2: Table of metabolites in low-salt shrimp paste, which were revealed by GC/MS machine, using ribitol as a standard, and performed in three replicates
