# Supplementary material for: Dynamic Changes in the Bacterial Community and Metabolic Profile during Fermentation of Low-Salt Shrimp Paste (Terasi)
Source: Metabolites. 2022 Jan 26;12(2):118. doi: 10.3390/metabo12020118 (PMC8874951; doi:10.3390/metabo12020118)
Supplement: Supplementary file 1 [file metabolites-12-00118-s001.zip › metabolites-1557528-supplementary.pdf]

Table S1. Table genus composition of shrimp and low- salt shrimp paste with abundancy more than 0.1% which revealed by Illumina sequencing using V3-V4 variable of 16S rRNA. T0=0 days of fermentation, T1=7 days of fermentation, T2=14 days of fermentation, T3=21 days of fermentation, T4=28 days of fermentation.

Genus	Shrimp	T0	T1	T2	T3	T4
Corynebacterium	0.17%	0.59%	1.01%	1.28%	0.99%	2.43%
Kocuria	0.03%	0.12%	0.12%	1.10%	0.11%	0.10%
Alkalibacillus	0.00%	12.01%	19.40%	16.73%*	15.62%*	15.11%
Lentibacillus	0.00%	1.44%	1.67%	4.23%	2.91%	1.86%
Oceanobacillus	0.00%	0.00%	0.01%	2.05%	0.01%	0.00%
Salimicrobium	0.00%	5.56%	5.64%	5.74%	4.91%	8.56%
Corticicoccus	0.00%	1.66%	1.50%	1.65%	1.82%	1.98%
Jeotgalicoccus	75.56%*	0.67%	0.62%	0.82%	1.43%	1.17%
Salinicoccus	22.01%	0.79%	0.72%	0.63%	0.95%	0.59%
Staphylococcus	0.33%	3.64%	2.73%	2.78%	3.74%	3.35%
Alkalibacterium	0.02%	8.87%	10.16%	9.04%	14.36%	19.80%
Alloiococcus	0.34%	22.44%	21.91%*	16.02%	13.55%	8.11%
Atopostipes	0.12%	10.80%	11.55%	11.17%	13.33%	22.52%*
Tetragenococcus	0.00%	23.46%*	16.20%	8.47%	12.01%	10.83%
Vibrio	0.18%	7.12%	5.72%	16.30%	13.13%	2.47%

Mark *in the same column showed three of the highest relative abundance of bacteria in the same of fermentation time

Table S2. Table of metabolites in low-salt shrimp paste which were revealed by GC/MS machine using ribitol as a standard and were done in three replicates.

Carbohydrate, purine/ pyrimidine	Carboxylic Acids	Amino acids	Fatty acids	Amine/amide	Phenolic	Other
**Mono, di-saccharides**	2-Picolinate	Alanine	Behenic acid (22:0)	1-3-Propanediamine	2-hydroxypiridine	2-aminoethanol
Glucose	Acetoacetic acid	Asparagine	Decanoic acid (10:0)	Cadaverine	4-hydroxybenzoic acid	Glycerol
Mannose	Allantoin	Aspartic acid	Elaidic acid (18-1n-9)	Histamine	4-hydroxymandelic acid	Phosphate
b-Lactose	Anthranilic acid	b-Alanine	Hepatdecanic acid (17:0)	n-Butylamine	4-hydroxyphenylpiruvic acid	
Maltose	Citramalic acid	b-Glutamic acid	Icosanoic acid (20:0)	n-Propylamine	4-hydroxyphenylacetic acid	
Ribose	Fumaric acid	Citrulline	Lauric acid (12:0)	Putrescine		
Sucrose	Gluconic acid	Cystathionine	Lignoceric acid (24:0)	Tryptamine		
N-acetyl glucosamine	Glutaric acid	Glutamic acid	Myristic acid (14:0)	Tyramine		
	Glyceric acid	Glutamine	Nonadecylic acid (19:0)	Urea		
**Alcoholic sugar**	Glycolic acid	Glycine	Nonanoric acid (9:0)			
Xylitol	Indole-3-acetic acid	Glycilglycine	Oleic acid(18:1n-9)			
Arabitol	Isocitric acid	Histidine	Palmitoleic acid (16:1n-7)			
Inositol	Lactic acid	Homocysteine	Pentadecanoic acid (15:0)			
Mannitol	Malic acid	Homoserine	Plamitic acid (16:0)			
	Methyl succinic acid	Hypotaurine	Stearic acid (17:0)			
**Purine/ pyrimidine**	Nicotinic acid	Isoleucine				
Adenine	Oxaloacetic acid	Pyroglutamic acid				
Guanine	Oxamic acid	Sarcosine				
Hypoxanthine	Prephenate	Serine				
Xanthine	Succinic acid	Taurine				
Guanosine	Uric acid	Threonine				
Inosine	Urocanate	Tryptophan				
2-Deoxyguanosine	Hydroxybutyric acid	Tyrosine				
2-Deoxyinosine	Hydroxyisovaleric acid	Valine				
Thymine		Hydroxyproline				

Uracil		2-Aminobutyric acid				
Uridine		2-Aminoadipic acid				
		3-Aminoisobutyric acid				
		4-aminobutyric acid				
Total=22	Total=24	Total=43	Total=15	Total =9	Total =5	Total =3
Total annotated metabolites = 121, Unannotataed metabolites = 577, Total metabolites = 698						
